# Machine learning analysis of serum cholesterol's impact on knee osteoarthritis progression

**DOI:** 10.1038/s41598-024-69906-2

**Published:** 2024-08-14

**Authors:** Hong-bo Li, Yong-jun Du, Guy Romeo Kenmegne, Cheng-wei Kang

**Affiliations:** 1 Department of Orthopaedics, The Third Affiliated Hospital of Guangxi Medical University, The Second People’s Hospital of Nanning City, Nanning, Guangxi China; 2 Department of Orthopaedics, West China Hospital, West China School of Medicine, Chengdu, Sichuan China; 3 West China School of Public Health and West China Fourth Hospital, Sichuan University, Chengdu, 610041 Sichuan China

**Keywords:** Knee osteoarthritis (KOA), Machine learning (ML), Model interpretation, Predictive modeling, Serum total cholesterol (TC), Epidemiology, Health care

## Abstract

The controversy surrounding whether serum total cholesterol is a risk factor for the graded progression of knee osteoarthritis (KOA) has prompted this study to develop an authentic prediction model using a machine learning (ML) algorithm. The objective was to investigate whether serum total cholesterol plays a significant role in the progression of KOA. This cross-sectional study utilized data from the public database DRYAD. LASSO regression was employed to identify risk factors associated with the graded progression of KOA. Additionally, six ML algorithms were utilized in conjunction with clinical features and relevant variables to construct a prediction model. The significance and ranking of variables were carefully analyzed. The variables incorporated in the model include JBS3, Diabetes, Hypertension, HDL, TC, BMI, SES, and AGE. Serum total cholesterol emerged as a significant risk factor for the graded progression of KOA in all six ML algorithms used for importance ranking. XGBoost algorithm was based on the combined best performance of the training and validation sets. The ML algorithm enables predictive modeling of risk factors for the progression of the KOA K–L classification and confirms that serum total cholesterol is an important risk factor for the progression of KOA.

## Introduction

Osteoarthritis (OA) is a very common degenerative disease and a major public health challenge globally, with osteoarthritis affecting more than 300 million people around the world^1^. OA is a major cause of pain, disability, and socioeconomic costs, with the knee joint being one of the most commonly affected areas^2^. Despite the high prevalence of knee osteoarthritis (KOA), there is still a lack of good and clinically effective medication for the clinical management of KOA to slow its progression or cure it. Currently, the most commonly used pharmacologic treatments for KOA include nonsteroidal anti-inflammatory drugs (NSAIDs). However, the long-term use of NSAIDs is associated with side effects such as gastrointestinal discomfort^3^, and the therapeutic effect of NSAIDs in severe osteoarthritis is still not satisfactory. Therefore, basic research on the pathogenesis of KOA and the search for clinical risk factors affecting disease progression is urgent.

Research on the pathogenesis of osteoarthritis has been overwhelming, with basic studies on cellular and molecular mechanisms, such as Zhang et al.^4^ who found that an imbalance in the macrophage ratio between pro-inflammatory (M1) and anti-inflammatory/tissue repair phenotypes (M2) plays a crucial role in the progression of OA. There have also been studies on the development of osteoarthritis through proteomics^5^. However, the clinical impact of basic research is "far-reaching", and it is difficult to have a direct effect on prevention and clinical intervention in the short term. There are also studies on the progression of KOA from metabolic factors, such as obesity, hypertension, diabetes, and dyslipidemia^6^. These aspects may be risk factors for the progression of osteoarthritis, and interventions in these areas may slow the progression of KOA.

The association between metabolic syndrome and KOA has been well-studied. By meta-analysis, Xie et al. found that metabolic syndrome and hypertension were positively associated with KOA, whereas dyslipidemia did not correlate^6^. A cross-sectional study by Dahaghin et al.^7^ found that overweight and diabetes mellitus were significantly associated with OA, hypertension was weakly associated with OA, and total cholesterol/high-density lipoprotein cholesterol ratio was not significantly associated with OA. However, other studies have shown a significant correlation between serum total cholesterol and bone and joints^8–11^. Given the current disagreement on whether serum total cholesterol is a risk factor for osteoarthritis, there is a need to further characterize the potential risk factors for osteoarthritis as early detection of these risk factors may block or slow the progression of KOA severity.

This study used data on KOA and related clinical information from the DRYAD public database to construct a prediction model through machine learning to analyze the risk factors affecting the progression of KOA and to provide clinicians with a clinical rationale for the early identification of these risk factors.

## Methods and materials

### Data sources

As this study was a retrospective secondary analysis based on data obtained from the open-access DRYAD database (https://datadryad.org/) the ethics committee waived the requirement for informed consent. The data were freely available for download and reuse with proper citation^12^ (Dryad dataset: 10.5061/dryad.79cnp5htv)) ^13^. The original data collection adhered to the principles set out in the Declaration of Helsinki and was approved by the Institutional Ethics Committee of Kasturba Medical College, Mangaluru (reference number IEC KMC MLR 10-18/363). Participants provided written informed consent at the time of the original study. In addition, case records of patients diagnosed with knee OA between January 2017 and September 2018 were accessed with permission from the medical superintendent of the hospitals associated with Kasturba Medical College, Mangalore, through the proper channels, including the head of the department of orthopedics and the Institutional Ethics Committee. Confidentiality was maintained by anonymizing the data, with each participant assigned a unique serial number.

### Subjects

The original study for this database was a cross-sectional study that included the participation of 225 patients. The researchers graded the severity of the patient's knee imaging according to the Kellgren and Lawrence (K–L) evaluation criteria^14^, of which 101 were graded K–L classification grade 2, 94 were graded K–L classification grade 3, and 30 were graded K–L classification grade 4. The database also incorporated participants' CVD risk factors (including age, body mass index, systolic blood pressure, diabetes mellitus, total cholesterol, high-density lipoprotein, and smoking) which were assessed. The JBS3 Risk Score Calculator was designed by the Joint British Societies of General Practitioners, and its primary role is to estimate a patient's lifetime risk of cardiovascular disease.

Inclusion criteria: ① Cases that passed the imaging grading K–L classification grade 2, 3, and 4; ② with complete clinical data; ③ patients' informed consent and voluntary participation in the study.

Exclusion criteria: ① 11 cases with a history of rheumatoid arthritis; ② patients who refused consent; ③ patients with secondary osteoarthritis of the knee; and ④ patients with known coronary artery disease were excluded from this study.

### Grouping definitions and data processing

For the purpose of binary logistic regression analysis, in this study, K–L classification grade 2 was defined as mild arthritis, and K–L classification grades 3 and 4 were defined as moderate-to-severe arthritis. Based on our definitions and inclusion and exclusion criteria, mild KOA was 101 cases and moderate-to-severe KOA was 113 cases in this study.

In the statistics, it was found that there are subgroups with counts of 0 in the four variables SES, AGE, Heart Age, and Life Expectancy, which cannot be used in the chi-square test. We will combine the SES: 1, 2, 3 sub-groups into one group; AGE: 6, 7 sub-groups are combined into one group; Heart Age: 8, 9 sub-groups are combined into one group; Life Expectancy: 4 and 5 sub-groups are combined into one group, 8 and 9 sub-groups are combined into one group, and then we will analyze the processed data again.

### Construction and evaluation of predictive models

The steps of predictive model construction are as follows: (1) Screening of characteristic factors: risk factors were screened for graded progression of KOA using LASSO regression analysis, adjusting the number of variables and complexity; (2) constructing a binary logistic regression model for univariate factor analysis, and observing the relationship between the screened characteristic variables and the dependent variable relationship between KLGrade, and at the same time, multifactorial analysis was conducted; the model visualization was obtained by applying the "rms" package to generate the Nomograms (version of R is: 4.2.3,version of rms is: 6.7.1); (3) To determine the important risk factors for the grading and progression of knee osteoarthritis, six machine learning (ML) algorithms were applied to rank the importance analysis of influencing factors, including logistic regression (LR), K nearest neighbor classification (KNN), extreme gradient boosting (XGBoost), random forest (RF), AdaBoost classifier(AB), and support vector machine classification (SVM), which were used to combine clinical features and related variables to build a prediction model; (4) Comprehensively based on the AUC ranking of the training set and validation set, selecting relatively good stability machine learning methods for classification, and generating a Shap-Value diagram of model interpretability, interpreting model importance and contribution through the Shap-Value diagram, and interpreting the model results by calculating the contribution of each feature to the prediction results. (5) To determine the optimal probability cutoffs for our classification models, we employed ROC curve analysis and also considered the Precision-Recall curve due to the class imbalance in our dataset. For both Logistic Regression and Random Forest models, we set the probability cutoff values based on the prevalence of the positive class in the training dataset, as recommended in the literature (https://www.mdpi.com/2078-2489/15/5/252). This method ensures that the cutoff values reflect the underlying data distribution, thereby optimizing model performance. Specifically, for the Logistic Regression model, we calculated the prevalence of the positive class in the training dataset and used this value as the cutoff. For instance, if the prevalence was 0.456, the probability threshold for classifying a case as positive was set to 0.456. Similarly, for the Random Forest model, the prevalence of the positive class was used to determine the cutoff. Aligning the cutoff values with the prevalence aims to balance sensitivity and specificity, thus enhancing the models' practical utility in clinical settings. The optimal cutoff values were further validated through cross-validation to ensure robustness. The optimal cutoff was further validated using cross-validation to ensure robustness. (Model interpretability Python version package description: SHAP 0.39.0).

### Statistical analysis

Categorical variables were counted as the number of categorical items, frequency/percentage, and comparisons of categorical variables were made using the chi-square test; continuous variables were counted as mean, median, 25%/75% quartile, and maximum and minimum values, and interquartile spacing was expressed using the (IQR), and comparisons were made by using the Mann–Whitney U test. Considering the small sample size of this study, risk factors for knee osteoarthritis were screened using LASSO regression analysis, which was performed using R software (version glmnet: 4.1.8). We assessed the statistical significance of our model performance using bootstrap methods. This involved resampling our dataset with replacement to create multiple training and testing sets, thereby estimating the variability of our performance metrics. Confidence intervals for the AUC and other performance metrics were calculated using the bootstrap samples. We also performed DeLong’s test to compare the AUCs of different models. A two-tailed test was used to determine the level of statistical difference, with *P* < 0.05 considered statistically significant. Statistical analysis was done using SPSS (version 22.0), R (version 4.2.3) and Python (version 3.7).

### Ethical declaration

As this study was a retrospective secondary analysis based on a publicly available dataset, the ethics committee waived the requirement for informed consent. Written informed consent was not required for this study according to national laws and the requirements of the research organization. Private information in the database has been anonymized.

## Results

### Baseline data

The total effective sample size of this study was 214 cases, The number of cases of mild KOA was 101 (KL Grade = 2, 47.20%); the number of cases of moderate to severe KOA was 113 (KL Grade = 3 + 4, 52.80%). Descriptive statistics for categorical and continuous variables are shown in Supplementary Tables S1, and comparative bar graph between the two groups are shown in Supplementary Fig. S1.

### Risk factor screening for risk of staging progression in KOA

LASSO regression is to fit a generalized linear model along with complexity adjustment to avoid overfitting and variable screening. LASSO regression performed risk factor screening on independent variables, and the λ of the smallest mean square error is 0.016, which corresponds to the variable selection of the model as JBS3, Diabetes, Hypertension, HDL, TC, BMI, SES, AGE, and the number of independent variables was reduced from 17 to 8. The coefficient curves and cross-validation curves for the LASSO regression are shown in Supplementary Fig. S2. The table of LASSO coefficients is shown in Supplementary Table S2.

### Univariate and multifactorial logistic regression analysis of KOA

**Figure 1:**
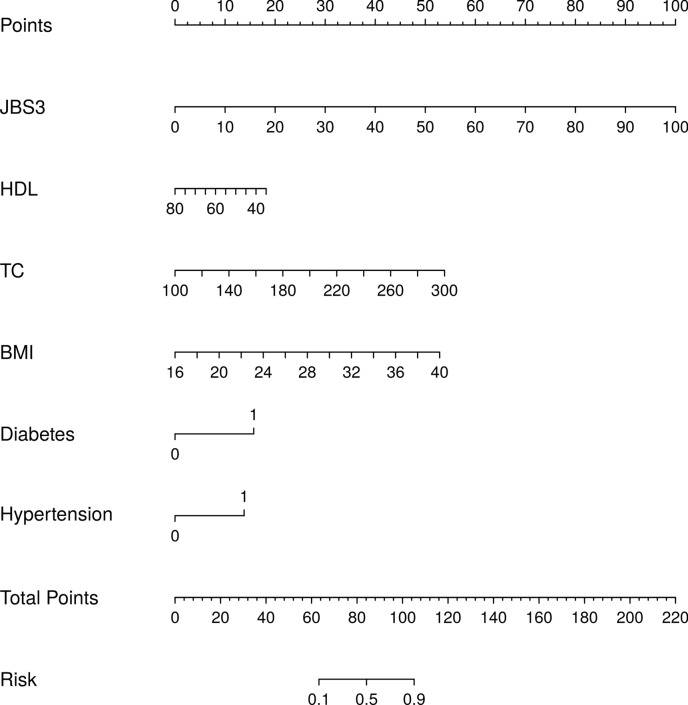
A Nomogram of the KOA imaging grading progression prediction model using these six variables. When using the nomogram, the position of the axes corresponding to each variable is first determined to derive the number of points corresponding to each predictor, and then the points of all predictors are summed. After the total number of points for all predictors is obtained, the predicted probability corresponding to the total number of points is the probability of KOA grading progression. **diabetes:** 1 = Yes, 0 = No; **Hypertension:** 1 = Yes, 0 = No.

To further control the effect of confounding factors, univariate and multivariate logistic regression analyses were performed on the eight risk factors screened by LASSO regression to observe the relationship between JBS3, HDL, TC, BMI, Diabetes, Hypertension, AGE, SES, and the dependent variable KL Grade. Furthermore, multivariate analyses were carried out, and the multivariate covariates of the model were GENDER, SMOKER, SBP (Table 1). The two variables of SES and AGE were further excluded (*P* > 0.05), and finally, the six factors of JBS3, HDL, TC, BMI, Diabetes, and Hypertension were obtained as the characteristic factors (*P* < 0.05), and the six variables were used to establish the KOA imaging grading progression prediction model, the forest plot is shown in Supplementary Fig. S3, and a nomogram was created using the "rms" software package (Fig. 1). The results of logistic regression analysis are shown in Supplementary Table S3.

**Table 1:** Unifactorial and multifactorial logistic regression analysis of KLGrade progression.

Variables	N	Univariable OR (95% CI)	*P*-value	Multivariable aOR (95% CI)	*P*-value_adjusted
JBS3	214.0	1.149 [1.102, 1.198]	0.000	1.166 [1.110, 1.225]	0.000
HDL	214.0	0.953 [0.917,0.991]	0.015	0.947 [0.907,0.988]	0.012
TC	214.0	1.037 [1.025,1.05]	0.000	1.032 [1.019,1.045]	0.000
BMI	214.0	1.259 [1.147,1.381]	0.000	1.205 [1.095,1.325]	0.000
Diabetes					
No	161.0			1 [Reference]	
Yes	53.0	1 [Reference]	0.000	15.713 [5.169,47.769]	0.000
Hypertension		18.566 [6.388, 53.963]			
No	118.0			1 [Reference]	
Yes	96.0	1 [Reference]	0.000	7.030 [3.536,13.976]	0.000
AGE		10.276 [5.380, 19.627]			
50–55	55.0			1 [Reference]	
56–60	56.0	1 [Reference]	0.502	0.854 [0.371,1.965]	0.711
61–65	52.0	1.292 [0.612,2.727]	0.392	1.317 [0.583,2.975]	0.509
66–70	33.0	1.395 [0.651,2.987]	0.038	2.088 [0.796,5.480]	0.135
71–75	11.0	2.583 [1.052,6.346]	0.090	3.214 [0.666,15.520]	0.146
76–85	7.0	3.444 [0.824,14.392]	0.503	0.801 [0.122,5.250]	0.817
SES		1.722 [0.352,8.437]			
1–3	62.0			1 [Reference]	
4	96.0	1 [Reference]	0.339	1.162 [0.556,2.427]	0.689
5	56.0	0.727 [0.378,1.398]	0.006	0.566 [0.249,1.286]	0.174
		0.354 [0.168,0.747]			

*OR*, odds ratio; *aOR*, adjusted odds ratio; *CI*, confidence interval. Variables in the multifactor logistic regression model were adjusted according to GENDER, SMOKER, and SBP.

The ROC curves and calibration curves of the logistic regression prediction model were plotted in R. The area under the ROC curve for the model is 0.93 (95% CI 0.897–0.963), which corresponds to the area under the ROC curve indicating that the model has a high degree of discrimination. The MAE of the standard curve was 0.039, which indicates that the model has a high degree of calibration. The ROC curve and the calibration curve of the logistic regression prediction model are shown in Supplementary Fig. S4.

### Impact factor importance analysis ranking

In this study, the final six characteristic factors of JBS3, HDL, TC, BMI, Diabetes, and Hypertension were ranked for the influence factor importance analysis using six machine learning methods, namely logistic regression (LR), K nearest neighbors classification (KNN), extreme gradient boosting (XGBoost), random forest (RF), AdaBoost Classifier (AB), and support vector machine classification (SVM).

**Figure 2:**
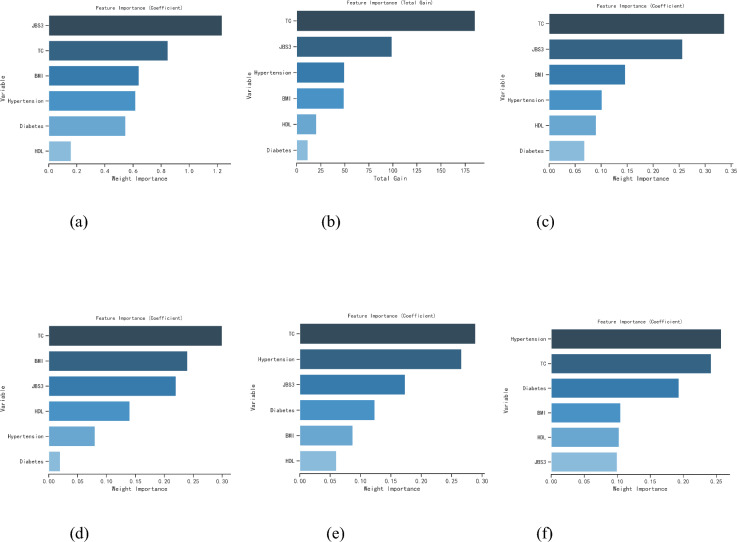
Schematics of the ranking of impact factor importance analysis using six machine learning methods. (**a**) logistic regression (LR); (**b**) extreme gradient boosting tree (XGBOOST); (**c**) random forrest classifier (RF); (**d**) AdaBoost classifier (AB); (**e**) support vector machine classification (SVM); (**f**) K nearest neighbor classification (KNN).

It can be seen that 'TC' is the first important risk factor in all four classification algorithms, namely, XGBOOST, RF, AB, and SVM, and is the second important risk factor in the two classification algorithms, namely, LR, and KNN algorithms are the second most important risk factors. The ranking of the six machine learning impact factors in terms of importance analysis is shown schematically in Fig. 2.

### Performance of machine learning algorithms

**Figure 3:**
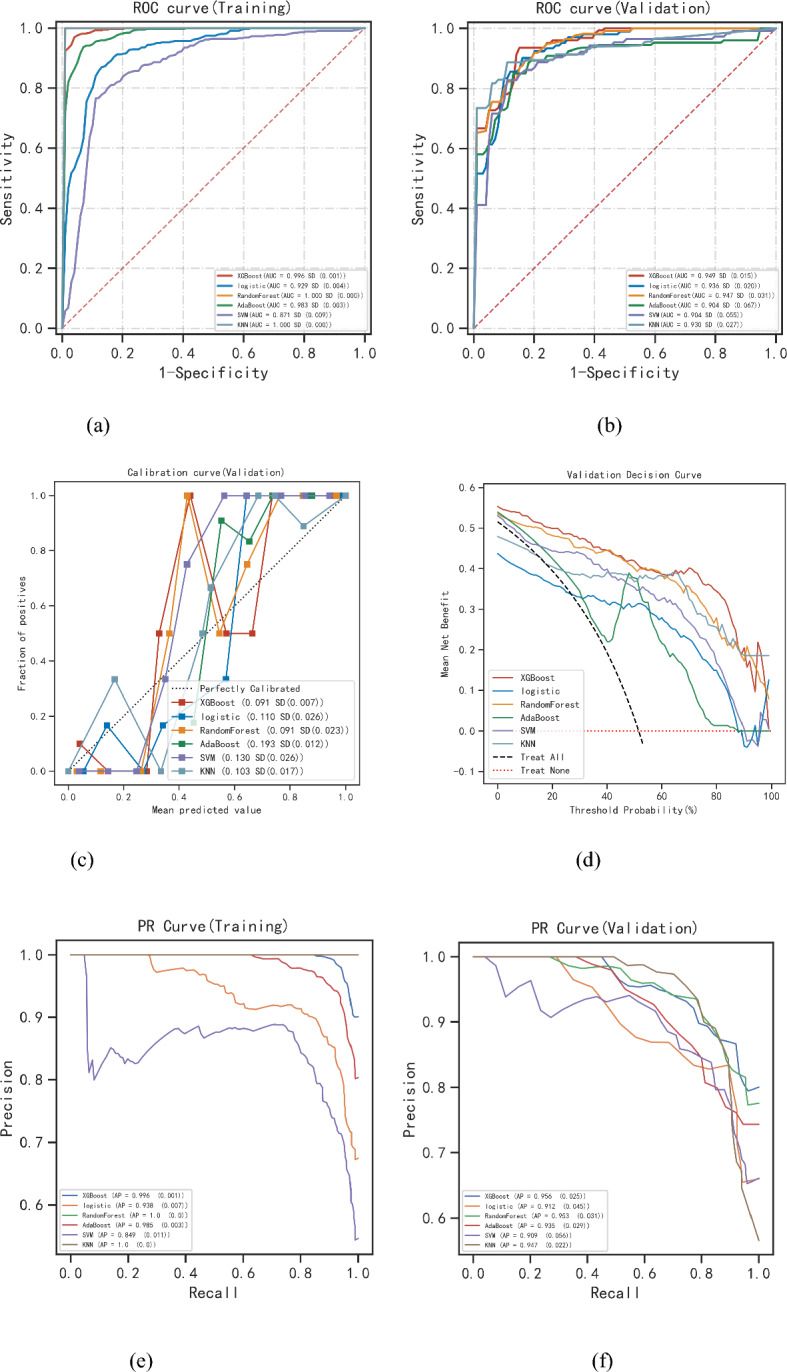
ML model synthesis analysis. (**a**) Training set ROC and AUC; (**b**) Test set ROC and AUC. Patients with KOA were sampled five times in an 8:2 ratio. (**c**) For the calibration curves of the test set, the horizontal coordinate is the average predicted probability, the vertical coordinate is the actual probability of the event, the diagonal dashed line is the reference line, and the other solid lines are the fitted lines of the different models. The closer the fitted line is to the reference line and the smaller the value in parentheses, the more accurate the model predictions are. (**d**) Test set DCA, where the black dashed line represents the assumption that all patients have severe osteoarthritis, and the red dashed line and the thin black line represent the assumption that patients do not have severe osteoarthritis. The remaining solid lines represent different models. (**e**, **f**) Training set, test set PR curves, and AP values. y-axis is precision and x-axis is recall.

Six machine learning models were used to complete the task of classifying the data samples. Variables in the models include JBS3, Hypertension, Diabetes, TC, HDL and BMI. The ROC results of KL Grade prediction for each model are shown in Fig. 3a and b, the ROC mean and SD of the models are cross-validated by fivefold, and the error lines in the figure are the ROC mean and SD. Among all the models so far, the best performer in the training set is random forest (Fig. 3a, based on the AUC ordering), and the best performer in the validation set is XGBoost (Fig. 3b, based on the AUC ordering). The calibration curves display that the XGB Classifier model has a higher prediction accuracy (Fig. 3c), DCA evaluates that the XGB Classifier is more suitable for clinical applications (Fig. 3d), and the XGB Classifier model has the highest AP value in the training and test sets (Fig. 3e and f). The AUC values of the XGBoost model are higher than those of the logistic regression model (DeLong test). Bootstrap analysis confirms the robustness of these results, with similar performance observed across multiple resampled datasets. The comprehensive analysis shows that the XGB Classifier can be considered the best model. The corresponding scores in the validation set for each evaluation criterion are shown in Table 2, and the corresponding scores in the training set for each evaluation criterion and the Z-value and P-value of delong detection are shown in Supplementary Table S4.

**Table 2:** Summary of results for multi-model classification-validation set.

Classification model	AUC(SD)	Cutoff (SD)	Accuracy (SD)	Sensitivity (SD)	Specificity (SD)	Positive predictive value (SD)	Negative predictive value (SD)	F1 score (SD)	Kappa (SD)
XGBoost	0.949 (0.015)	0.484 (0.096)	0.870 (0.024)	0.900 (0.073)	0.927 (0.061)	0.881 (0.055)	0.869 (0.070)	0.887 (0.040)	0.729 (0.048)
logistic	0.936 (0.020)	0.456 (0.067)	0.842 (0.071)	0.906 (0.026)	0.892 (0.068)	0.774 (0.165)	0.923 (0.044)	0.823 (0.097)	0.687 (0.138)
RF	0.947 (0.031)	0.626 (0.035)	0.865 (0.031)	0.869 (0.091)	0.911 (0.067)	0.934 (0.064)	0.809 (0.032)	0.898 (0.062)	0.731 (0.062)
AB	0.904 (0.067)	0.498 (0.003)	0.833 (0.047)	0.886 (0.111)	0.869 (0.078)	0.897 (0.059)	0.783 (0.099)	0.886 (0.065)	0.664 (0.086)
SVM	0.904 (0.055)	0.560 (0.019)	0.819 (0.040)	0.863 (0.066)	0.880 (0.055)	0.887 (0.089)	0.763 (0.028)	0.875 (0.076)	0.638 (0.082)
KNN	0.930 (0.027)	1.000 (0.000)	0.502 (0.067)	0.868 (0.050)	0.959 (0.039)	NaN (NaN)	0.502 (0.067)	NaN (NaN)	0.000 (0.000)

*LR* Logistic Regression; *KNN* K Nearest Neighbor Classification; *XGBoost* Extreme Gradient Boosting; *RF* Random Forest; *AB* AdaBoost Classifier; *SVM* Support Vector Machine Classification.

### Optimal model construction and evaluation

Based on the AUC ordering of the training set and validation set, Random Forest (RF) is highly likely to have an overfitting phenomenon, and XGBoost stability is relatively good, so we use the XGB Classifier machine learning method for classification. The XGB Classifier machine learning method was used to classify the variables in the model as KL Grade: JBS3, HDL, TC, BMI, Diabetes, and Hypertension.

The test set of N = 42 cases (20.00%) was randomly selected from the overall sample, and the remaining samples were used as the training set for fivefold cross-validation, and the final model obtained AUC = 0.9335 ± 0.0175 in the validation set. The AUC = 0.9661 in the test set, and the accuracy = 0.9302. Given that the performance of the AUC metrics in the validation set did not exceed that of the test set or the ratio was less than 10% (Supplementary Fig. S5), it can be assumed that the model fit was successful and the XGBoost model can be used for the classification modeling task for this dataset. The baseline characteristics of the training and validation sets are shown in Supplementary Table S5.

### From SHAP to model interpretation

**Figure 4:**
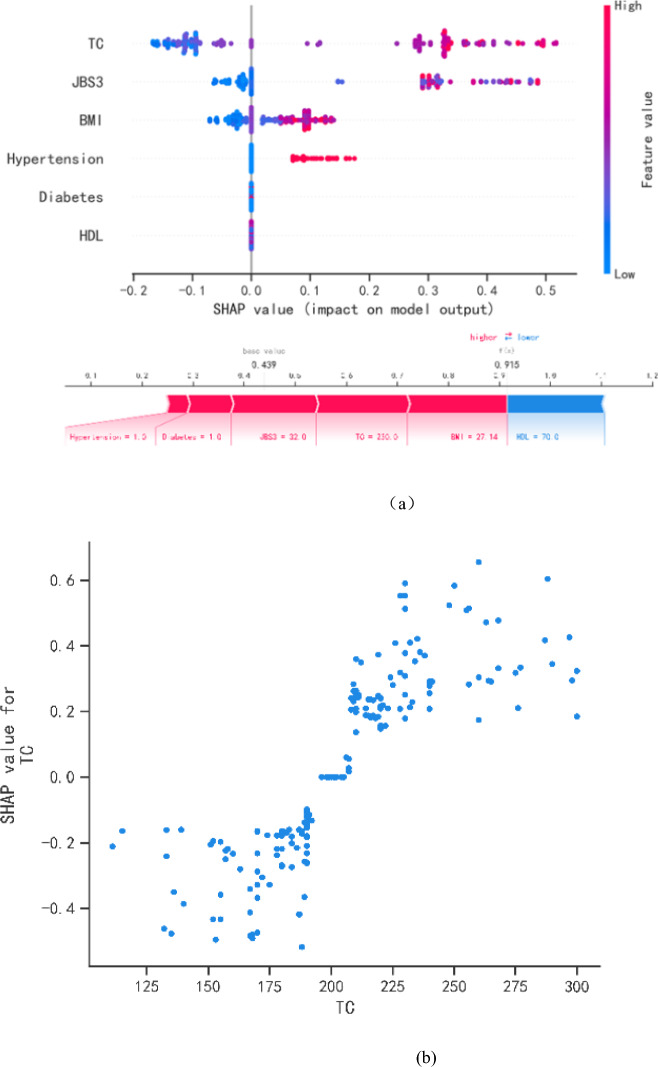
SHAP interpretation model. (**a**) Attributes of features in SHAP. Each line represents a feature and the horizontal coordinate is the SHAP value. Red dots indicate higher feature values and blue dots indicate lower feature values. SHAP values represent the predictive features of individual patients and the contribution of each patient to the predictive progress. The length of the arrows helps to visualize how much the prediction is affected. The longer the arrow, the greater the effect. (**b**) Dot plot of the correlation between the "risk factor TC" and the distribution of the scatterplot, which shows that the risk of KOA progression increases after a TC greater than 200 mg/dl.

We use SHAP Value diagram to visualize the covariance of each variable in the selected variable prediction model on the predicted outcomes. Figure 4a shows the six most important features of the model and predictive features and their contribution to the prediction of progression. On each significant feature line, all patient attributions to the outcome are plotted with different colored dots, where red dots represent high-risk values and blue dots represent low-risk values. Elevated TC, JBS3, BMI, Diabetes, and Hypertension increase a patient's risk of KOA progression, whereas elevated HDL decreases the risk of progression, and can be considered a KOA protective factor. The high SHAP values for TC indicate its strong influence on the progression of knee osteoarthritis. The risk factor TC correlation point plots are shown in Fig. 4b. (XGBoost: python version description: xgboost 1.2.1, model interpretability python version description: shap 0.39.0).

## Discussion

This is a cross-sectional study based on the DRYAD public database, which was used by Yue et al. to conduct a clinical model of knee osteoarthritis progression, with very clinically relevant results^15^. However, there were several shortcomings in their study, for example, Yue et al.'s study excluded patients with KL 4 from the database, which is an important classification for KOA, the lack of such an important variable may have an impact on the final accuracy of the results; furthermore, the study did not include patients with a history of rheumatoid arthritis in the database as part of their exclusion criteria, having known that rheumatoid arthritis is a significant risk factor for KOA; Rheumatoid arthritis can therefore be considered as a significant confounder. The current study was optimized on the previous study by including all cases of KL 2, 3, and 4 in the data analysis and excluding patients with a history of diagnosed rheumatoid arthritis, therefore, our study may have done a better job of controlling confounders; moreover the accuracy of the model may have been superior.

Early assessment of risk factors for staged progression in patients with KOA is critical, and if variables that have a value in influencing outcomes are identified from the multitude of risk factors, those will be the key to intervention. The variables in the original database are numerous because it is not possible to determine which factors have a greater impact on the target variables at the beginning of the database creation, but too many variables are detrimental to our early intervention in the clinic. To compress and filter variables from the numerous variables, we used LASSO regression and one-way logistic regression analysis to filter six characteristic variables from 17 clinical variables and used these six streamlined variables to construct a predictive model to assess the risk of progression of knee osteoarthritis imaging grading. This method was chosen to minimize overfitting and enhance the interpretability of the predictive models. LASSO regression selects variables that are most strongly associated with the outcome, thereby simplifying the model without sacrificing predictive power. However, we acknowledge that this approach may introduce biases as highlighted in Chen et al.^16^, which discusses ethical machine learning in healthcare. In our study, elevated TC, JBS3, BMI, Diabetes, and Hypertension increased the risk of progression of KOA in patients, whereas elevated HDL decreased the risk of progression. We used six common machine learning methods, Logistic Regression (LR), K nearest neighbor classification (KNN), extreme gradient boosting (XGBoost), random forest (RF), AdaBoost classifier (AB), and support vector machine classification (SVM) to rank the importance of the impact factor analyses. It can be concluded that 'TC' is in XGBOOST, RF, AB, and SVM, which are the four classification algorithms, and 'TC' is the first-ranked important risk factor, and the second most important risk factor in the two algorithms, LR and KNN. So, in our study, 'TC' was a significant risk factor for the progression of K–L classification in KOA imaging.

In such a study, we not only confirmed TC, JBS3, BMI, Hypertension, and Diabetes as risk factors for the graded progression of KOA but also found HDL as a protective factor for the graded progression of KOA imaging, which is another valuable conclusion obtained in this study.

There are many clinical risk factors for the progression of KOA severity, and identifying key risk factors can help guide clinical prevention. In this study, we used six ML models, and after analyzing the AUC, DCA, calibration curves, and PR curves, although the RF model was the best performer in the training set among all the models (ranked based on AUC), the combined performance of the XGBClassifier model was overall better than that of the other ML models, and so we chose the XGBClassifier as the best model. Finally, we use SHAP-Value diagram to visually explain the important weights of the XGBClassifier predictive model and variable factors.

The XGB Classifier was first proposed as an algorithm by Chen et al.^17^. In recent years this algorithm has been very popular. It is a boosting algorithm based on Gradient Boosting Classifier, XGB classifier is an integrated classifier that uses regularization techniques to reduce the overfitting problem and this method is superior to gradient boosting algorithm. It improves the performance of the integrated classification algorithm by generating stronger models from many weaker models using an iterative approach, the advantage of XGB classifier is that it performs well when compared to Gaussian Plain Bayes, Decision Tree and Random Forest classifiers, the disadvantage of XGB classifier is that it requires more CPU execution time to classify the data^18^.

In this study, a machine learning algorithm was used to conclude that serum total cholesterol is an important risk factor for the progression of KOA. Hypercholesterolemia is a systemic metabolic disorder characterized by abnormally elevated blood cholesterol levels, which affects not only the cardiovascular system and internal organs but also the musculoskeletal system^19^. Previous studies have identified hypercholesterolemia as a risk factor for rotator cuff injury and glenohumeral osteoarthritis^20,21^, Hypercholesterolemia has also been shown to be associated with the progression of KOA^22^, and the mechanism may be related to the fact that hypercholesterolemia alters the expression of A Disintegrin and Metalloprotease with Thrombospondin Motifs ADAMTS and Matrix Metalloproteinases (MMPs) in the synovial membrane of the knee, which are mainly found in the joints. MMP-1 is predominantly found in synoviocytes within joints, and the family of matrix metalloproteinase (MMP)-producing and deintegrin and metalloproteinase structural domains with platelet-responsive protein motifs (ADAMTS) plays a key role in extracellular matrix disruption in musculoskeletal disorders such as arthritis^23^.

### Limitations of this study

Our study has several limitations. Firstly, this is a cross-sectional study based on a public database, and further prospective studies are needed to confirm the reliability of our model; secondly, the data was collected at a single institution with small sample size and clinical variables, and we may have omitted important predictors that may be more clinically valuable in the progression of KOA, which requires the construction of larger study centers with larger sample size and database of clinical variables for optimization, we are also aware of the potential bias in variable selection using LASSO regressions; again, there may be a risk of incomplete exclusion criteria for osteoarthritis in this database, such as possible comorbidities with other types of arthritis (e.g., gouty arthritis, etc.), and so the generalizability of the results of this study is limited. Finally, although a high degree of consistency was achieved in the reproducibility analyses within the training and test sets, some unavoidable errors may occur due to segmentation uncertainty. Longitudinal studies or prospective case–control studies are also needed to further explain the relationship between risk factors and the progression of KOA imaging severity.

## Conclusions

In summary, this study constructed a prediction model based on the ML model, and the XGB Classifier model showed better performance in the study. In addition, we also visualized with a SHAP-Value diagram of KOA patients with imaging grading progression provided a personalized risk assessment, and confirmed that TC is an important risk factor. This study may help clinicians and patients to identify the risk of KOA progression at an early stage, and interfering with the risk factors may potentially help to slow or block the progression of KOA.

### Supplementary Information

Supplementary Information.

## Data Availability

Data are available upon request from the corresponding author, KCW.

## Abbreviations

IQR: Interquartile range
OR: Odds ratio
aOR: Adjusted odds ratio
CI: Confidence interval
ROC: Receiver operator characteristic
AUC: Area under the ROC curve
KL Grade: Grade of knee osteoarthritis as per Kellgren Lawrence classifiction
SES: Socio economic status of the patient as per the B G Prasad scale (1: Upper, 2: Upper Middle, 3: Lower Middle, 4: Upper Lower, 5: Lower)
CVD: Whether the patient has a past history of cardiovascular disease
Diabetes: Whether the patient has diabetes mellitis
Hypertension: Whether the patient is currently on any antihypertensive treatment
Heart Age: Physiological heart age of the patient calculated as per JBS3 risk score calculator
Life Expectancy: Life expectancy of the patient calculated as per the JBS3 risk score calculator
Height: Height of the patient in centimeters
Weight: Weight of the patient in kilograms
BMI: Body mass index of the patient in kilograms per square meter
Heart Age: Physiological heart age of the patient calculated as per patient in kilograms per square meter
TC: Serum total cholesterol of the patient in milligram per deciliter
HDL: Serum high density lipoprotein of the patient in milligram per deciliter patient in milligram per deciliter
SBP: Systolic blood pressure of the patient in millimeters of mercury
JBS3: Percent risk of developing cardiovascular disease in the next 10 years calculated as per the JBS3 risk score calculator
