# Supplementary material for: Machine learning analysis of serum cholesterol's impact on knee osteoarthritis progression
Source: Sci Rep. 2024 Aug 14;14:18852. doi: 10.1038/s41598-024-69906-2 (PMC11324727; doi:10.1038/s41598-024-69906-2)
Supplement: Supplementary file 1 — Supplementary Information. [file 41598_2024_69906_MOESM1_ESM.docx]

**Serum total cholesterol: an important risk factor for progression of knee osteoarthritis**

-- Establishment and validation of machine learning prediction models

（**Supplementary materials**）

Hong-bo Li, MD^a^, Yong-jun Du, MD^a^, Cheng-wei Kang, MD^b,*^ , Guy Romeo Kenmegne, MD^c^

*^a^Department of Orthopaedics, The Third Affiliated Hospital of Guangxi Medical University/The Second People's Hospital of Nanning City, Nanning, Guangxi, China*

*^b^West China School of Public Health and West China Fourth Hospital, Sichuan University, Chengdu, Sichuan, China*

*^c^Department of Orthopaedics, West China Hospital, West China School of Medicine, Chengdu, Sichuan, China*

*Corresponding author:*

*Address correspondence to Chengwei Kang, M.D., Department of Orthopedics, West China School of Public Health and West China Fourth Hospital, Sichuan University, Chengdu 610041, Sichuan, China. E-mail: kangarooqq@163.com,* [*Tel:(+86)-18683934660*](Tel:(+86)-18683934660)

**Supplementary Table S1(a)** Baseline characteristics in the mild and moderate-severe KOA groups

| Variables | Sub-  group | Total number  (n=214) | Mild KOA  (KL Grade2, n=101) | Moderate to severe KOA  (KL Grade3+4, n=113) | Statistic | P-value |
| --- | --- | --- | --- | --- | --- | --- |
| GENDER ,n(%) | Female | 124 (57.944) | 57 (56.436) | 67 (59.292) | 0.179 | 0.673 |
|  | Male | 90 (42.056) | 44 (43.564) | 46 (40.708) |  |  |
| AGE ,n(%) | 50-55 | 55 (25.701) | 31 (30.693) | 24 (21.239) | 6.397 | 0.269 |
|  | 56-60 | 56 (26.168) | 28 (27.723) | 28 (24.779) |  |  |
|  | 61-65 | 52 (24.299) | 25 (24.752) | 27 (23.894) |  |  |
|  | 66-70 | 33 (15.421) | 11 (10.891) | 22 (19.469) |  |  |
|  | 71-75 | 11 (5.140) | 3 (2.970) | 8 (7.080) |  |  |
|  | 76-85 | 7 (3.271) | 3 (2.970) | 4 (3.540) |  |  |
| SES ,n(%) | 1-3 | 62 (28.972) | 23 (22.772) | 39 (34.513) | 8.023 | 0.018 |
|  | 4 | 96 (44.860) | 43 (42.574) | 53 (46.903) |  |  |
|  | 5 | 56 (26.168) | 35 (34.653) | 21 (18.584) |  |  |
| SMOKER ,n(%) | No | 173 (80.841) | 85 (84.158) | 88 (77.876) | 1.359 | 0.244 |
|  | Yes | 41 (19.159) | 16 (15.842) | 25 (22.124) |  |  |
| CVD ,n(%) | No | 180 (84.112) | 96 (95.050) | 84 (74.336) | 17.122 | <0.001 |
|  | Yes | 34 (15.888) | 5 (4.950) | 29 (25.664) |  |  |
| Diabetes, n (%) | No | 161 (75.234) | 97 (96.040) | 64 (56.637) | 44.438 | <0.001 |
|  | Yes | 53 (24.766) | 4 (3.960) | 49 (43.363) |  |  |
| Hypertension ,n(%) | No | 118 (55.140) | 83 (82.178) | 35 (30.973) | 56.530 | <0.001 |
|  | Yes | 96 (44.860) | 18 (17.822) | 78 (69.027) |  |  |
| Heart Age ,n(%) | 50-55 | 30 (14.019) | 26 (25.743) | 4 (3.540) | 66.008 | <0.001 |
|  | 56-60 | 19 (8.879) | 13 (12.871) | 6 (5.310) |  |  |
|  | 61-65 | 40 (18.692) | 26 (25.743) | 14 (12.389) |  |  |
|  | 66-70 | 33 (15.421) | 20 (19.802) | 13 (11.504) |  |  |
|  | 71-75 | 22 (10.280) | 6 (5.941) | 16 (14.159) |  |  |
|  | 76-80 | 18 (8.411) | 5 (4.950) | 13 (11.504) |  |  |
|  | 81-85 | 17 (7.944) | 3 (2.970) | 14 (12.389) |  |  |
|  | 86-95 | 35 (16.355) | 2 (1.980) | 33 (29.204) |  |  |
| Life Expectancy ,n(%) | 66-75 | 26 (12.150) | 5 (4.950) | 21 (18.584) | 17.179 | <0.001 |
|  | 76-80 | 64 (29.907) | 26 (25.743) | 38 (33.628) |  |  |
|  | 81-85 | 98 (45.794) | 51 (50.495) | 47 (41.593) |  |  |
|  | 86-95 | 26 (12.150) | 19 (18.812) | 7 (6.195) |  |  |
| HEIGHT ,median[IQR] |  | 163.000[155.000,170.000] | 160.000[154.000,170.000] | 163.000[157.000,170.000] | -1.554 | 0.120 |
| WEIGHT ,median[IQR] |  | 69.000 [60.000, 78.000] | 65.000 [60.000, 70.000] | 73.000 [66.000, 80.000] | -5.223 | <0.001 |
| BMI ,median[IQR] |  | 25.920 [23.430, 28.300] | 24.200 [22.850,27.040] | 27.300 [24.340, 29.410] | -5.129 | <0.001 |
| TC ,median[IQR] |  | 205.000[184.000,228.000] | 188.000 [170.000, 201.000] | 220.000 [208.000, 234.000] | -8.261 | <0.001 |
| HDL ,median[IQR] |  | 52.000 [47.000, 58.000] | 53.800 [50.000, 59.000] | 51.000 [45.000, 56.000] | 2.422 | 0.015 |
| SBP ,median[IQR] |  | 130.000[122.000,150.000] | 126.000 [120.000, 130.000] | 140.000 [130.000, 150.000] | -6.259 | <0.001 |
| JBS3 ,median[IQR] |  | 14.000 [9.300, 26.000] | 10.000 [6.000,13.000] | 23.000 [15.000, 36.000] | -8.294 | <0.001 |

**Note:** Categorical variables were compared using the chi-square test and continuous variables using the Mann-Whitney U test*.SES, GENDER, AGE, SMOKER, CVD, Diabetes, Hypertension, WEIGHT, BMI, TC, HDL, SBP,* and *JBS3* were tested using the Mann-whitney U test. IQR, interquartile range.

**Explanation:** **KL** Grade=Grade of KOA as per Kellgren-Lawrence Classification; **SES**= Socio economic Status of the patient as per the B G Prasad scale (1= Upper, 2= Upper Middle, 3= Lower Middle, 4= Upper Lower, 5= Lower); **CVD**=Whether the patient has a past history of cardiovascular disease. **Diabetes**=Whether the patient has Diabetes Mellitus; **Hypertension**=Whether the patient is currently on any antihypertensive treatment; **Heart Age**= Physiological Heart Age of the patient calculated as per JBS3 risk score calculator; **Life Expectancy**=Life Expectancy of the patient calculated as per the JBS3 risk score calculator; **Height**= Height of the patient in centimeters; **Weight**= Weight of the patient in kilograms; **BMI**= Body Mass Index of the patient in kilograms per square meter; **Heart Age**= Physiological Heart Age of the patient calculated as per patient in kilograms per square meter; **TC**=Serum Total Cholesterol of the patient in milligram per deciliter; **HDL**=Serum High-density Lipoprotein of the patient in milligram per deciliter patient in milligram per deciliter; **SBP**=Systolic Blood Pressure of the patient in millimeters of mercury; **JBS3**=Percent risk of developing cardiovascular disease in the next 10 years calculated as per the JBS3 risk score calculator.

**Supplementary Table S1(b)** Descriptive Statistics for Categorical Variables

| variant | categorized items | frequency |
| --- | --- | --- |
| AGE | 7 | 56 |
| GENDER | 2 | 124 |
| SES | 5 | 96 |
| SMOKER | 2 | 173 |
| Hypertension | 2 | 118 |
| Diabetes | 2 | 161 |
| CVD | 2 | 180 |
| HeartAge | 9 | 40 |
| Life Expectancy | 6 | 98 |
| KL Grade(biclassification) | 2 | 113 |
| KL Grade of Knee OA | 3 | 101 |

**Supplementary Table S1(c)** Continuous Variables Descriptive Statistics

| variant | average number | upper quartile | 25% quartile | 75% quartile | (statistics) standard deviation | minimum value | maximum values |
| --- | --- | --- | --- | --- | --- | --- | --- |
| HEIGHT | 162.827 | 163.000 | 155.000 | 170.000 | 8.582 | 142.000 | 182.000 |
| WEIGHT | 69.112 | 69.000 | 60.000 | 78.000 | 10.676 | 46.000 | 106.000 |
| BMI | 26.204 | 25.960 | 23.430 | 28.258 | 3.681 | 17.400 | 38.560 |
| TC | 205.921 | 205.500 | 184.000 | 227.500 | 35.505 | 111.000 | 300.000 |
| HDL | 52.434 | 52.000 | 47.250 | 58.000 | 7.306 | 38.000 | 77.000 |
| SBP | 135.794 | 130.000 | 122.500 | 149.500 | 17.879 | 100.000 | 190.000 |
| JBS3 | 19.606 | 14.000 | 9.475 | 26.000 | 15.285 | 3.000 | 94.000 |

**Supplementary Table** **S2** LASSO Coefficients Table

| name | coef |
| --- | --- |
| (Intercept) | -6.457 |
| Life Expectancy | 0.0 |
| JBS3 | 0.041 |
| Heart Age | 0.002 |
| CVD | 0.0 |
| Diabetes | 0.635 |
| Hypertension | 0.909 |
| SBP | 0.0 |
| HDL | 0.0 |
| TC | 0.016 |
| SMOKER | 0.0 |
| BMI | 0.081 |
| WEIGHT | 0.0 |
| HEIGHT | 0.0 |
| SES | 0.0 |
| GENDER | 0.0 |
| AGE | 0.0 |

**Supplementary Table** **S3** Logistic Regression Analysis Results Table

| Predictor | Estimate | SE | Z | p | Odds Ratio | Lower | Upper |
| --- | --- | --- | --- | --- | --- | --- | --- |
| (Intercept) | -14.221 | 2.427 | -5.859 | 0.0 | 0.0 | 0.0 | 0.0 |
| JBS3 | 0.105 | 0.027 | 3.908 | 0.0 | 1.111 | 1.057 | 1.175 |
| TC | 0.029 | 0.007 | 4.425 | 0.0 | 1.03 | 1.017 | 1.044 |
| BMI | 0.225 | 0.064 | 3.515 | 0.0 | 1.252 | 1.112 | 1.432 |
| Diabetes1 | 1.581 | 0.656 | 2.409 | 0.016 | 4.858 | 1.444 | 19.895 |
| Hypertension1 | 1.416 | 0.449 | 3.151 | 0.002 | 4.119 | 1.725 | 10.142 |

In this study, binary logistic regression was used to assess the effects of JBS3, HDL, TC, BMI, Diabetes, and Hypertension on KL Grade. The AUC of the training data was 0.93, and the model predicted well. Among the variables included in the model, the coefficient of the variable JBS3 was 0.105, OR was 1.111, and the p-value was 0, which was significant, implying that there will be an impact relationship on the outcome.

The coefficient of variable TC is 0.029, OR is 1.03, the p-value is 0 which is significant and means that there will be an impact relationship on the outcome. The coefficient of variable BMI is 0.225, OR is 1.252, and the p-value is 0 which is significant and means that there will be an impact relationship on the outcome. The coefficient of variable Diabetes1 is 1.581, OR is 4.858, p-value is 0.016 which is significant and means that there will be an impact relationship on the outcome. The coefficient of variable Hypertension1 is 1.416, the OR value is 4.119, p-value is 0.002, which is significant and means that there will be an impact relationship on the outcome. The results of the logistic regression analysis are shown in Supplementary **Table 4**. Cox-Snell R2 is 0.665, and Nagelkerke R2 is 0.499. R version is 4.2.3, the major packages involved are pROC 1.18.4, rms 6.7.1, forestploter 6.7.1, and the main packages involved are pROC 1.18.4, rms 6.7.1, and forestploter 6.7.1. Forestploter: 1.1.1, dplyr: 1.1.3, MASS: 7.3.60.

**Supplementary Table S4(a)** Multi-model Classification - Summary of Training Set Results

| classification model | AUC(SD) | cutoff(SD) | Accuracy (SD) | Sensitivity (SD) | Specificity (SD) | Positive predictive value (SD) | Negative predictive value (SD) | F1 score (SD) | Kappa (SD) |
| --- | --- | --- | --- | --- | --- | --- | --- | --- | --- |
| XGBoost | 0.996 (0.001) | 0.484 (0.096) | 0.964 (0.007) | 0.976 (0.017) | 0.964 (0.020) | 0.966 (0.019) | 0.961 (0.019) | 0.971 (0.006) | 0.927 (0.014) |
| logistic | 0.929 (0.004) | 0.456 (0.067) | 0.876 (0.011) | 0.895 (0.042) | 0.863 (0.032) | 0.890 (0.014) | 0.864 (0.033) | 0.891 (0.016) | 0.749 (0.019) |
| Random Forest | 1.000 (0.000) | 0.626 (0.035) | 0.994 (0.000) | 1.000 (0.000) | 1.000 (0.000) | 1.000 (0.000) | 0.988 (0.000) | 1.000 (0.000) | 0.988 (0.000) |
| AdaBoost | 0.983 (0.003) | 0.498 (0.003) | 0.933 (0.010) | 0.942 (0.010) | 0.936 (0.024) | 0.942 (0.022) | 0.925 (0.006) | 0.942 (0.010) | 0.866 (0.019) |
| SVM | 0.871 (0.009) | 0.560 (0.019) | 0.830 (0.007) | 0.804 (0.036) | 0.871 (0.029) | 0.874 (0.015) | 0.792 (0.020) | 0.837 (0.014) | 0.661 (0.013) |
| KNN | 1.000 (0.000) | 1.000 (0.000) | 0.464 (0.017) | 1.000 (0.000) | 1.000 (0.000) | NaN (NaN) | 0.464 (0.017) | NaN (NaN) | 0.000 (0.000) |

**Supplementary Table S4(b)** Delong detection Z-values Mean values Table

| name | XGBClassifier | LogisticRegression | RandomForestClassifier | AdaBoostClassifier | SVC | KNeighborsClassifier |
| --- | --- | --- | --- | --- | --- | --- |
| XGB Classifier | NA | 4.76 | 5.429 | 4.141 | 5.532 | 5.221 |
| Logistic Regression | 4.76 | NA | 0.435 | 0.775 | 0.882 | 0.682 |
| Random Forest Classifier | 5.429 | 0.435 | NA | 0.799 | 1.12 | 0.639 |
| AdaBoost Classifier | 4.141 | 0.775 | 0.799 | NA | 0.92 | 0.825 |
| SVC | 5.532 | 0.882 | 1.12 | 0.92 | NA | 0.845 |
| KNeighbors Classifier | 5.221 | 0.682 | 0.639 | 0.825 | 0.845 | NA |

**Supplementary Table S4(c)** Table of P-value means for the delong test

| Model | XGBClassifier | LogisticRegression | RandomForestClassifier | AdaBoostClassifier | SVC | KNeighborsClassifier |
| --- | --- | --- | --- | --- | --- | --- |
| XGB Classifier | NA | 0.0 | 0.0 | 0.0 | 0.0 | 0.0 |
| Logistic Regression | 0.0 | NA | 0.667 | 0.475 | 0.451 | 0.513 |
| Random Forest Classifier | 0.0 | 0.667 | NA | 0.538 | 0.427 | 0.553 |
| AdaBoost Classifier | 0.0 | 0.475 | 0.538 | NA | 0.493 | 0.436 |
| SVC | 0.0 | 0.451 | 0.427 | 0.493 | NA | 0.402 |
| KNeighbors Classifier | 0.0 | 0.513 | 0.553 | 0.436 | 0.402 | NA |

**Supplementary Table S5** Baseline Characterization of Training and Validation Sets

| variant | categorized items | Overview  (n=214) | Training set  0 (n=172) | Validation set  1 (n=42) | statistic | p |
| --- | --- | --- | --- | --- | --- | --- |
| AGE ,n(%) | 50-55 | 55 (25.701) | 46 (26.744) | 9 (21.429) | 9.492 | 0.091 |
|  | 56-60 | 56 (26.168) | 51 (29.651) | 5 (11.905) |  |  |
|  | 61-65 | 52 (24.299) | 38 (22.093) | 14 (33.333) |  |  |
|  | 66-70 | 33 (15.421) | 24 (13.953) | 9 (21.429) |  |  |
|  | 71-75 | 11 (5.140) | 7 (4.070) | 4 (9.524) |  |  |
|  | 76-85 | 7 (3.271) | 6 (3.488) | 1 (2.381) |  |  |
| GENDER ,n(%) | Female | 124 (57.944) | 103 (59.884) | 21 (50.000) | 1.353 | 0.245 |
|  | Male | 90 (42.056) | 69 (40.116) | 21 (50.000) |  |  |
| SES ,n(%) | 1-3 | 62 (28.972) | 49 (28.488) | 13 (30.952) | 1.391 | 0.499 |
|  | 4 | 96 (44.860) | 75 (43.605) | 21 (50.000) |  |  |
|  | 5 | 56 (26.168) | 48 (27.907) | 8 (19.048) |  |  |
| SMOKER ,n(%) | No | 173 (80.841) | 140 (81.395) | 33 (78.571) | 0.174 | 0.677 |
|  | Yes | 41 (19.159) | 32 (18.605) | 9 (21.429) |  |  |
| Hypertension ,n(%) | No | 118 (55.140) | 100 (58.140) | 18 (42.857) | 3.187 | 0.074 |
|  | Yes | 96 (44.860) | 72 (41.860) | 24 (57.143) |  |  |
| Diabetes, n (%) | No | 161 (75.234) | 130 (75.581) | 31 (73.810) | 0.057 | 0.811 |
|  | Yes | 53 (24.766) | 42 (24.419) | 11 (26.190) |  |  |
| CVD ,n(%) | No | 180 (84.112) | 147 (85.465) | 33 (78.571) | 1.200 | 0.273 |
|  | Yes | 34 (15.888) | 25 (14.535) | 9 (21.429) |  |  |
| Heart Age ,n(%) | 50-55 | 30 (14.019) | 27 (15.698) | 3 (7.143) | 12.136 | 0.096 |
|  | 56-60 | 19 (8.879) | 16 (9.302) | 3 (7.143) |  |  |
|  | 61-65 | 40 (18.692) | 33 (19.186) | 7 (16.667) |  |  |
|  | 66-70 | 33 (15.421) | 30 (17.442) | 3 (7.143) |  |  |
|  | 71-75 | 22 (10.280) | 18 (10.465) | 4 (9.524) |  |  |
|  | 76-80 | 18 (8.411) | 13 (7.558) | 5 (11.905) |  |  |
|  | 81-85 | 17 (7.944) | 13 (7.558) | 4 (9.524) |  |  |
|  | 86-95 | 35 (16.355) | 22 (12.791) | 13 (30.952) |  |  |
| Life Expectancy ,n(%) | 66-75 | 26 (12.150) | 22 (12.791) | 4 (9.524) | 2.868 | 0.412 |
|  | 76-80 | 64 (29.907) | 47 (27.326) | 17 (40.476) |  |  |
|  | 81-85 | 98 (45.794) | 81 (47.093) | 17 (40.476) |  |  |
|  | 86-95 | 26 (12.150) | 22 (12.791) | 4 (9.524) |  |  |
| KL Grade ,n(%) | Mild | 101 (47.196) | 83 (48.256) | 18 (42.857) | 0.395 | 0.530 |
|  | Moderate to severe | 113 (52.804) | 89 (51.744) | 24 (57.143) |  |  |
| HEIGHT ,median[IQR] |  | 163.000 [155.000, 170.000] | 163.000 [155.000, 170.000] | 163.000 [155.000, 170.000] | -0.249 | 0.804 |
| WEIGHT ,median[IQR] |  | 69.000 [60.000, 78.000] | 70.000 [60.000, 78.000] | 66.000 [61.000, 74.000] | 1.298 | 0.194 |
| BMI ,median[IQR] |  | 25.920 [23.430, 28.300] | 26.120 [23.730, 28.400] | 24.500 [22.500, 27.400] | 1.686 | 0.092 |
| TC ,median[IQR] |  | 205.000 [184.000, 228.000] | 205.000 [180.000, 225.000] | 210.000 [190.000, 230.000] | -1.590 | 0.112 |
| HDL ,median[IQR] |  | 52.000 [47.000, 58.000] | 52.000 [48.000, 58.000] | 53.000 [46.000, 58.000] | -0.270 | 0.788 |
| SBP ,median[IQR] |  | 130.000 [122.000, 150.000] | 130.000 [122.000, 148.000] | 134.000 [126.000, 150.000] | -1.241 | 0.212 |
| JBS3 ,median[IQR] |  | 14.000 [9.300, 26.000] | 13.000 [8.100,24.000] | 20.000 [10.000, 35.000] | -2.406 | 0.016 |

To analyze whether the difference of each index in the grouping of the training set and validation set of the study is statistically significant, the total effective sample is 214 cases, of which the training set: number of cases is 172; the validation set: the number of cases is 42. AGE, GENDER, SES, SMOKER, Hypertension, Diabetes, CVD, Heart Age, Life Expectancy, KL Grade by chi-square test p>0.05, no statistically significant difference between groups; JBS3 by Mann-Whitney U test p-value<0.05, the statistically significant difference between groups.IQR, interquartile range.

**Supplementary Figures**


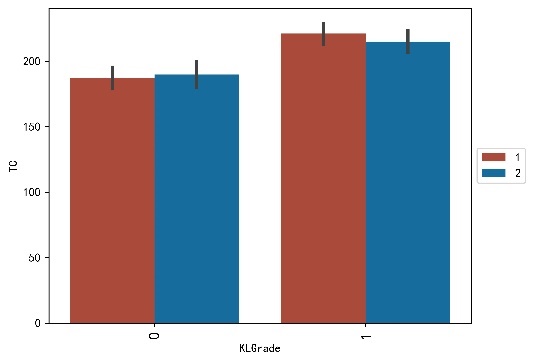

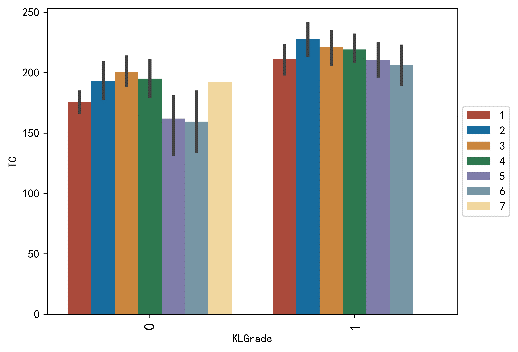


(a) GENDER (b) AGE


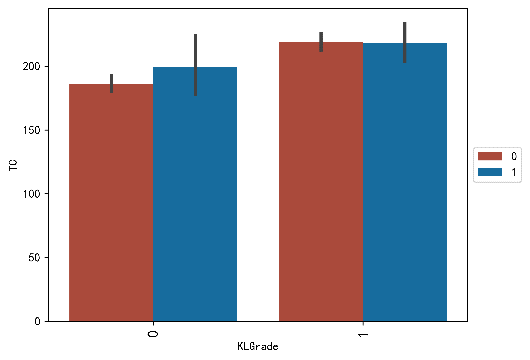

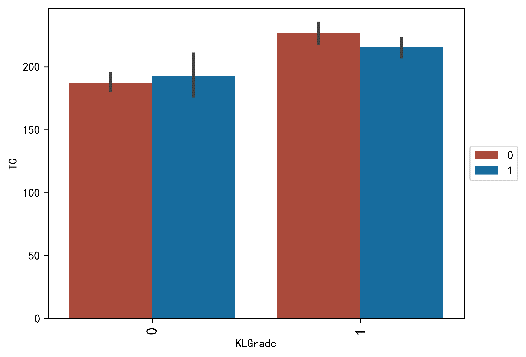


(c) SMOKER (d) HPERTENTION


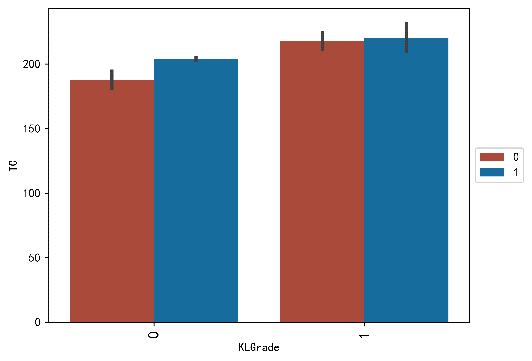

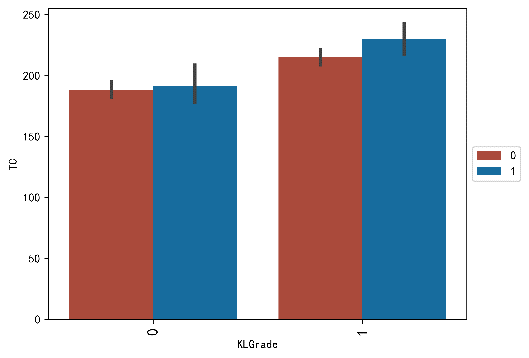


(e) Diabetes (f) CVD


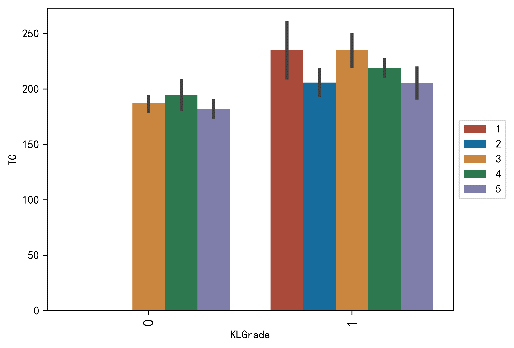

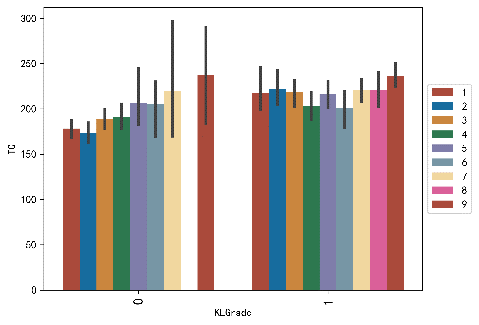


(g) SES (h) heart age

**Supplementary Fig.S1** Comparative bar graph between the two groups. 0= Mild KOA, 1= Moderate to severe KOA.

**KL** Grade=Grade of Knee Osteoarthritis as per Kellgren lawrence Classifiction; (a) Gender=Gender of the Patient (1= Female, 2=Male) ; (b)Age= Age of the patient (1= 50-55, 2= 56-60, 3= 61-65, 4=66-70, 5=71-75, 6=76-80, 7=81-85); (c)Smoker=Whether the patient is smoker or not (1=Yes, 0=No)**;** (d) Hypertension=Whether the patient is currently on any antihypertensive treatment (1=Yes, 0=No); (e)Diabetes=Whether the patient has Diabetes Mellitis(1=Yes, 0=No); (f)CVD=Whether the patient has a history of cardiovascular disease(1=Yes, 0=No); (g)SES= Socio economic Status of the patient as per the B G Prasad scale (1= Upper, 2= Upper Middle, 3= Lower Middle, 4= Upper Lower, 5= Lower); (h) Heart Age=Physiological Heart Age of the patient calculated as per JBS3 risk score calculator (1= 50-55, 2= 56-60, 3= 61-65, 4=66-70, 5=71-75, 6=76-80, 7=81-85, 8=86-90, 9=91-95).


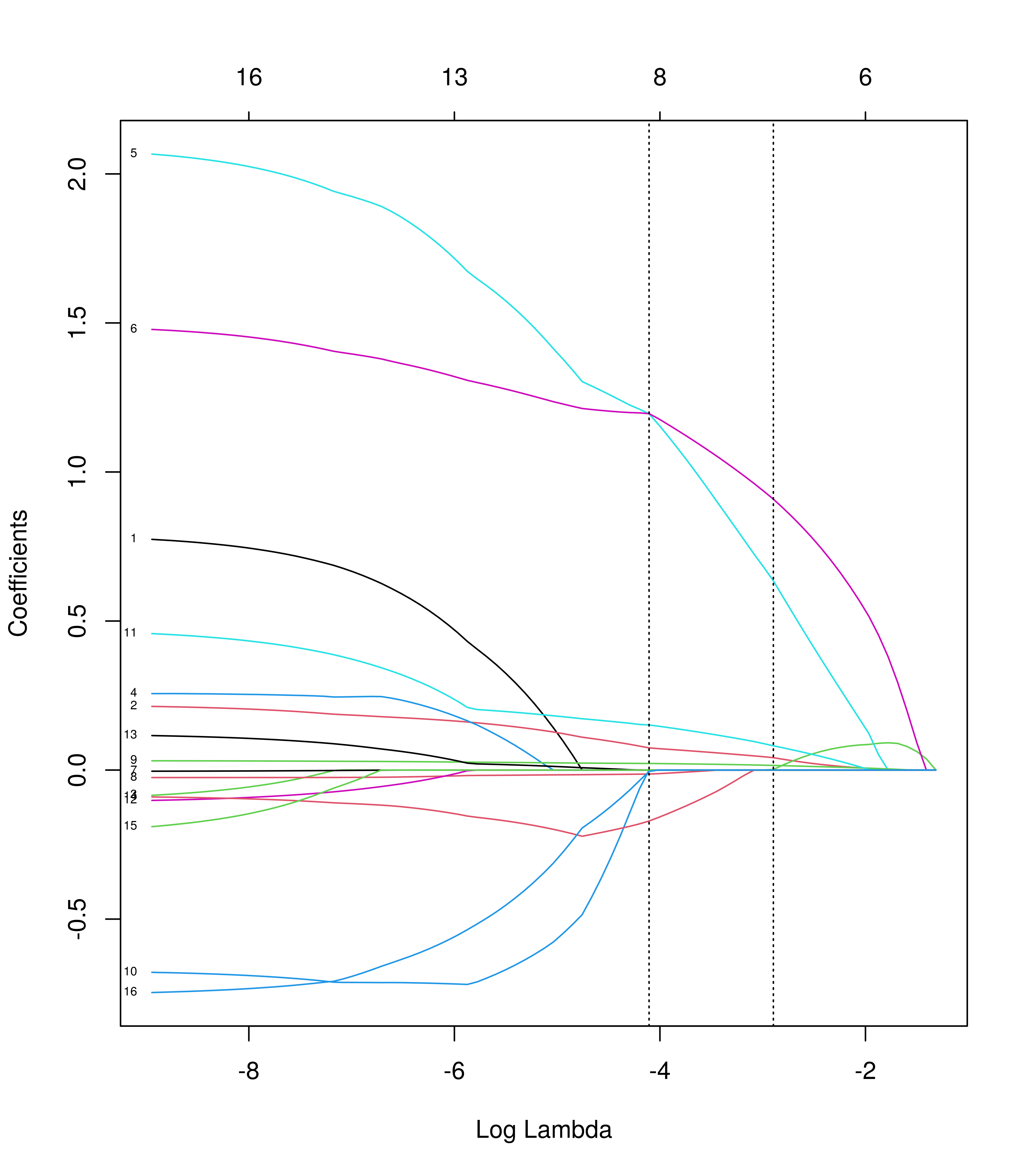

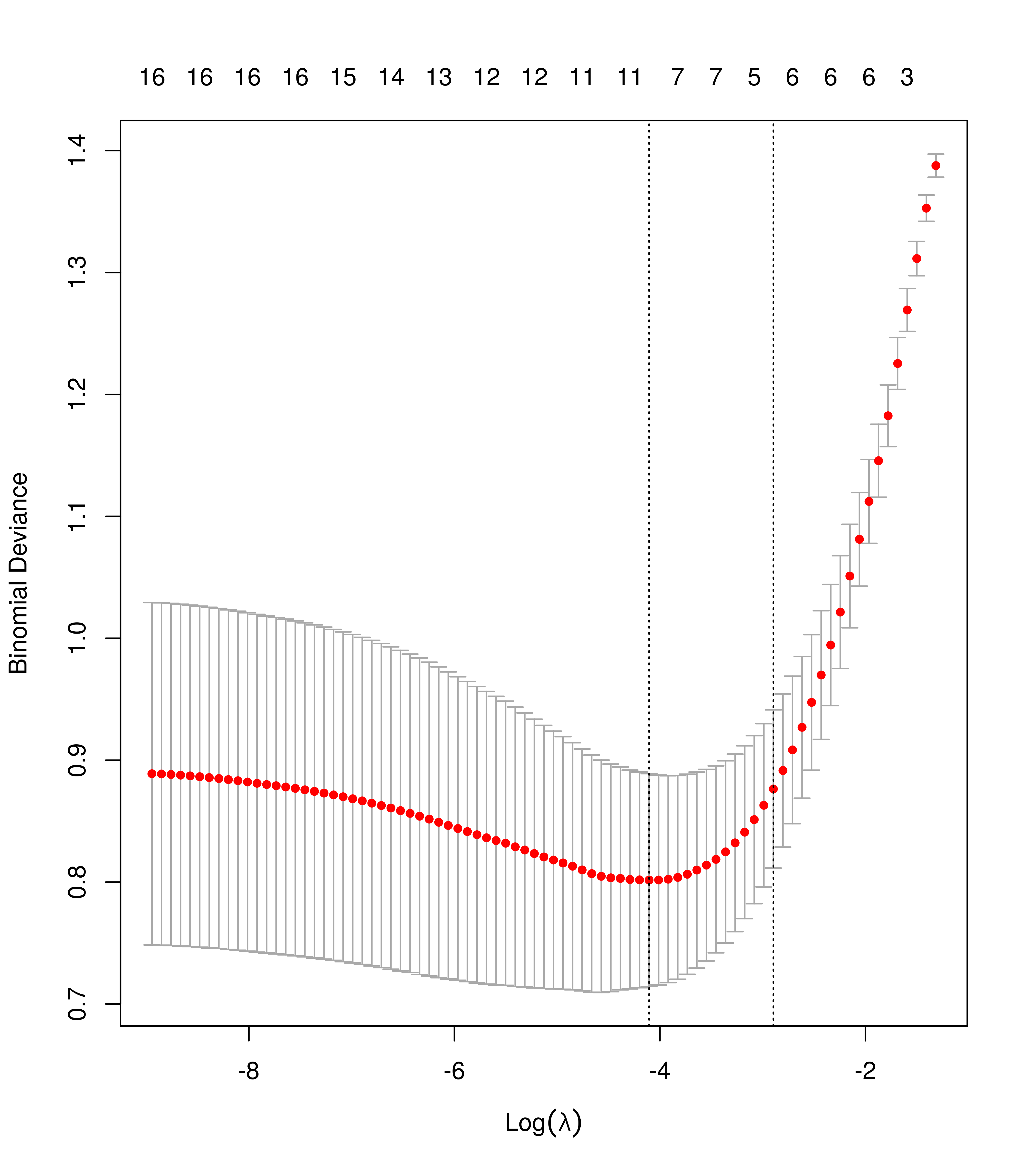


1. (b)

**Supplementary Fig.S2.** Feature factor selection using LASSO regression analysis. (a) Coefficient profiles for 16 texture features from the log (λ) sequence in the LASSO model are plotted. Vertical dashed lines are drawn at the minimum mean square error (λ = 0.016) and the minimum distance standard error (λ = 0.055). (b) Vertical lines are plotted at selected values using 5-fold cross-validation where the optimal lambda yields 8 nonzero coefficients.


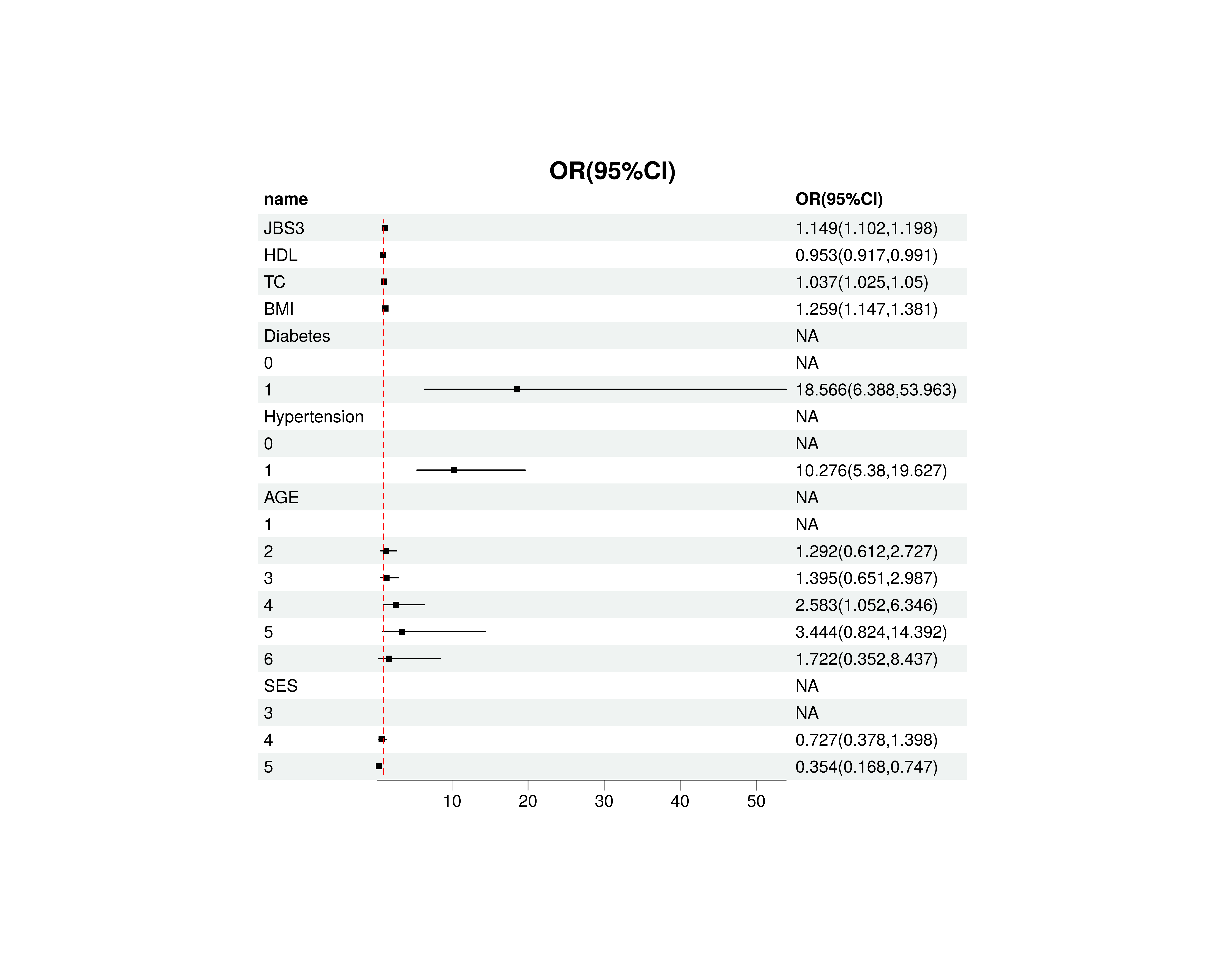


(a)


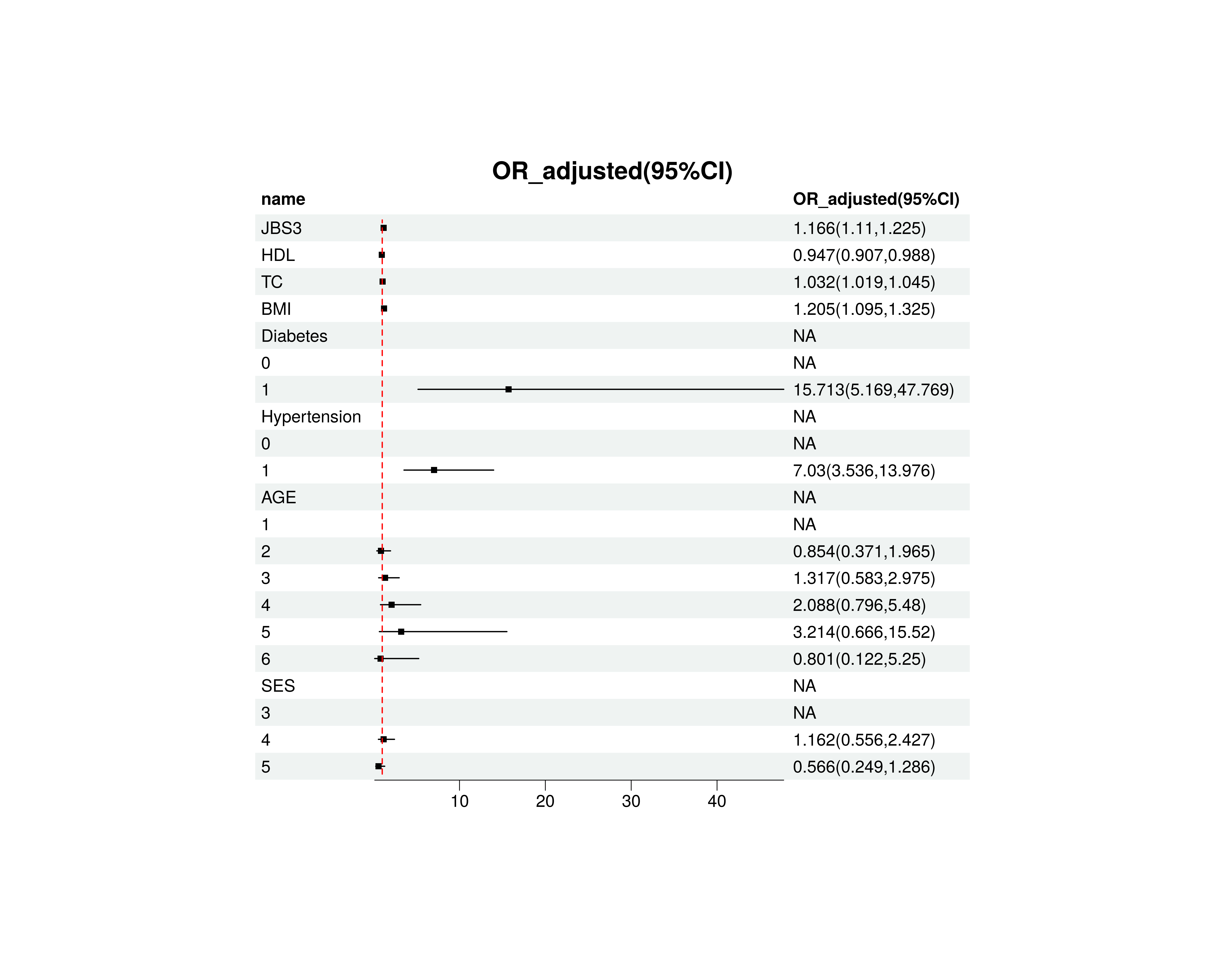


(b)

**Supplementary Fig.S3.** Forest plot for logistic regression. (a) Forest plot for one-factor logistic regression analysis; (b) Forest plot for multifactor logistic regression analysis.


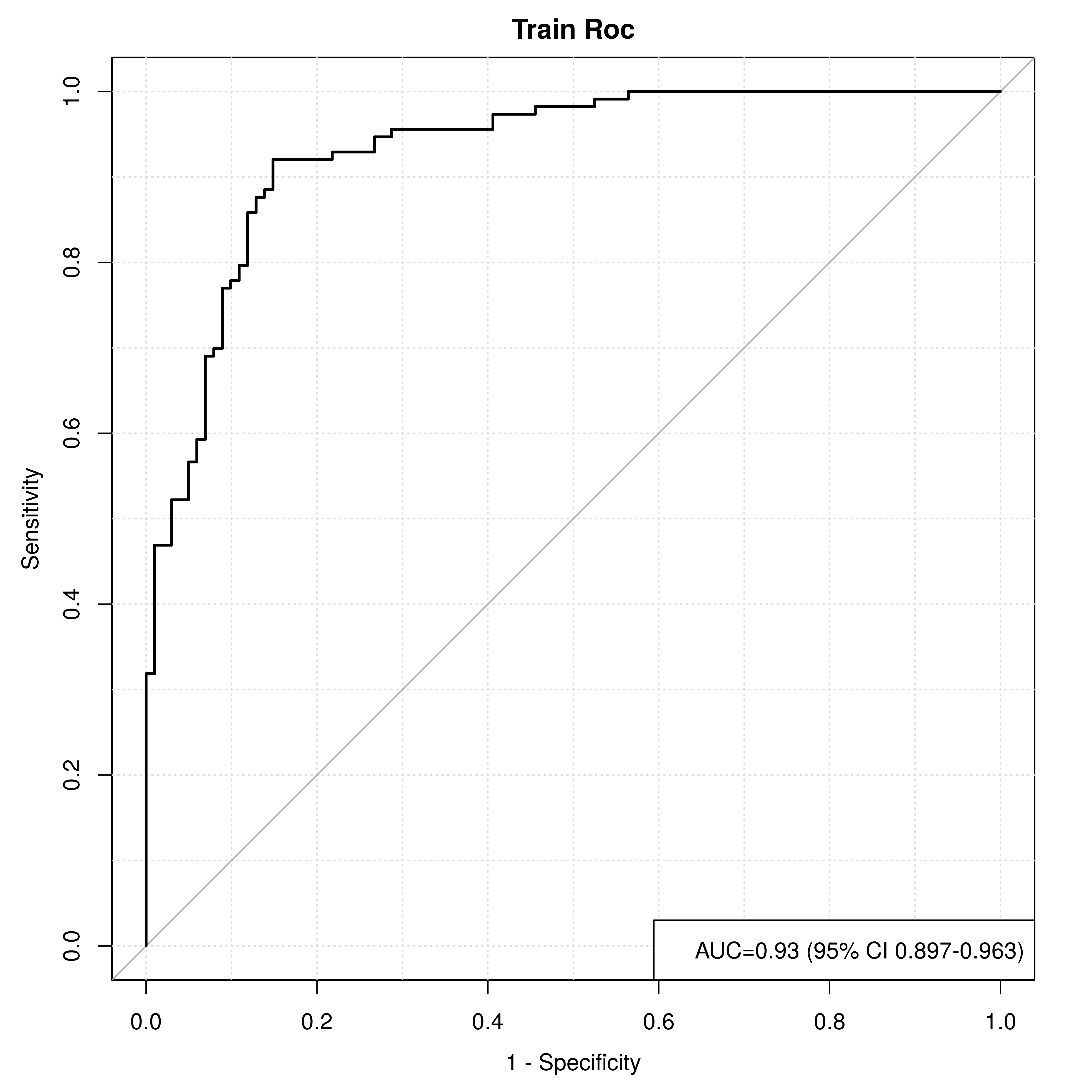

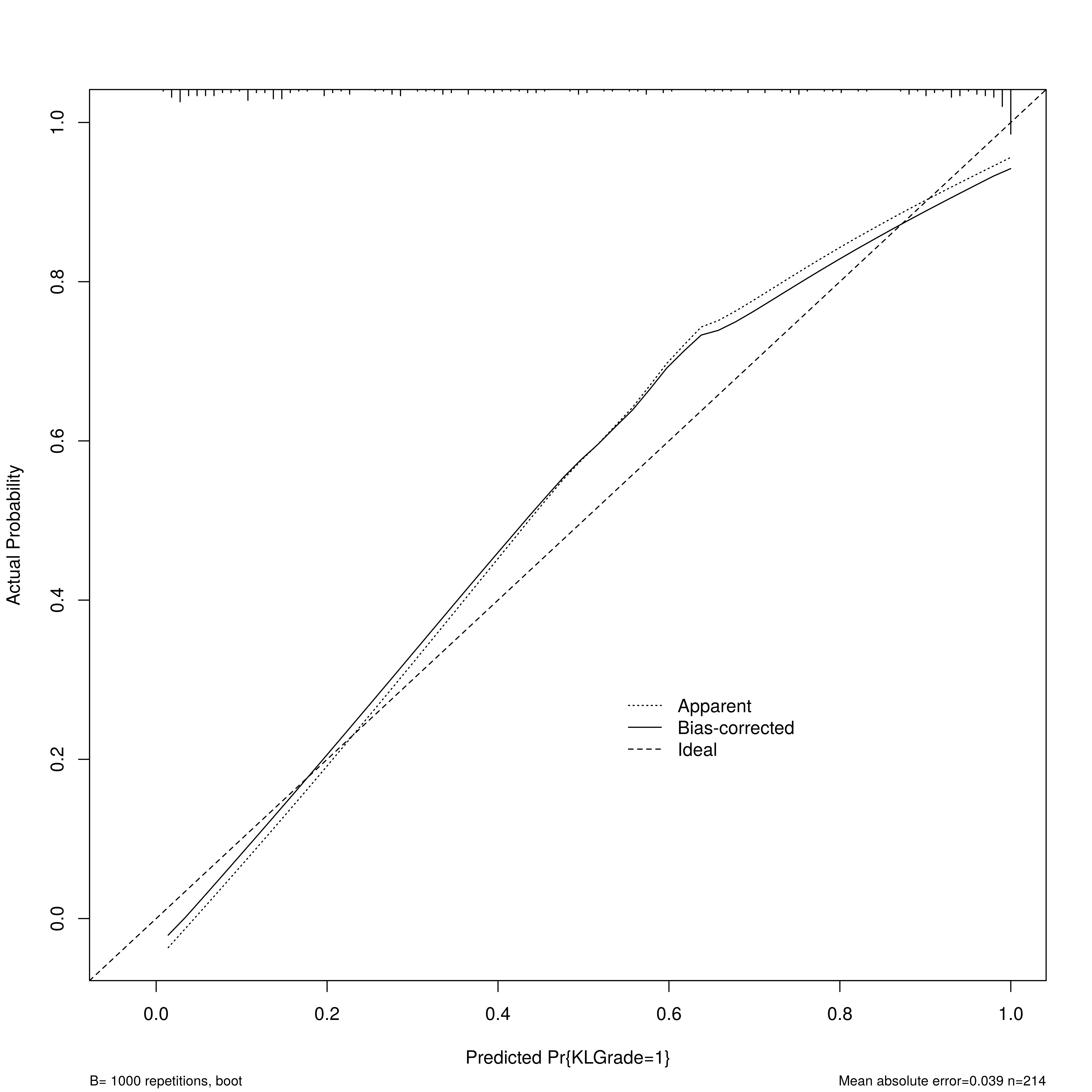


1. (b)

**Supplementary Fig.S4** ROC curve and a calibration curve of logistic regression prediction model. (a) ROC curve of the logistic regression prediction model, the area under the ROC curve is 0.93 (95% CI 0.897-0.963), which indicates that the model has a better discriminatory degree; (b) calibration curve of the logistic regression prediction model.


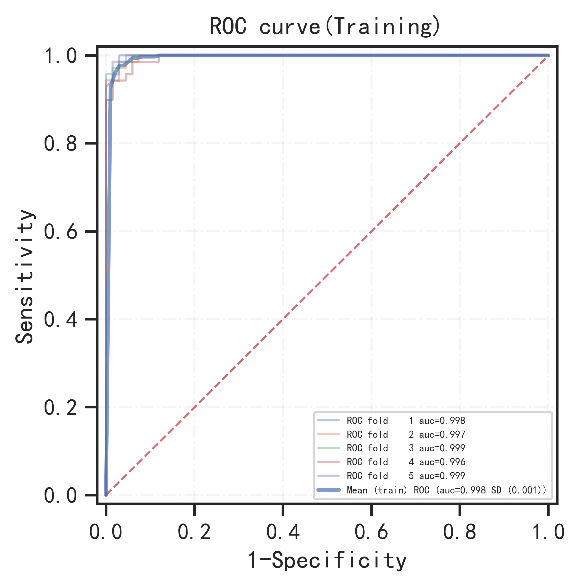

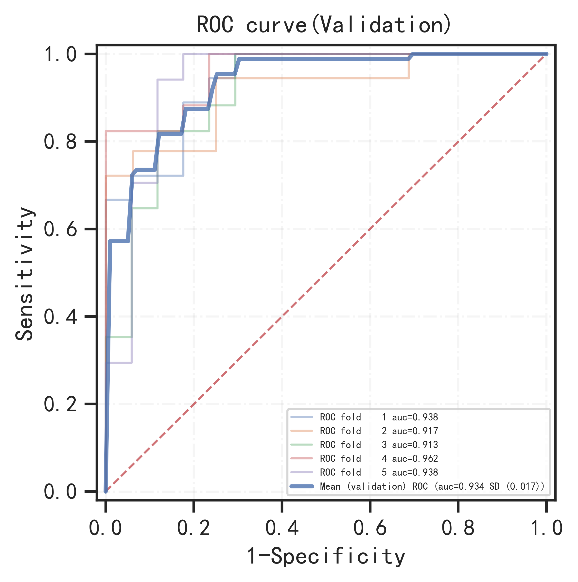


(a) (b)


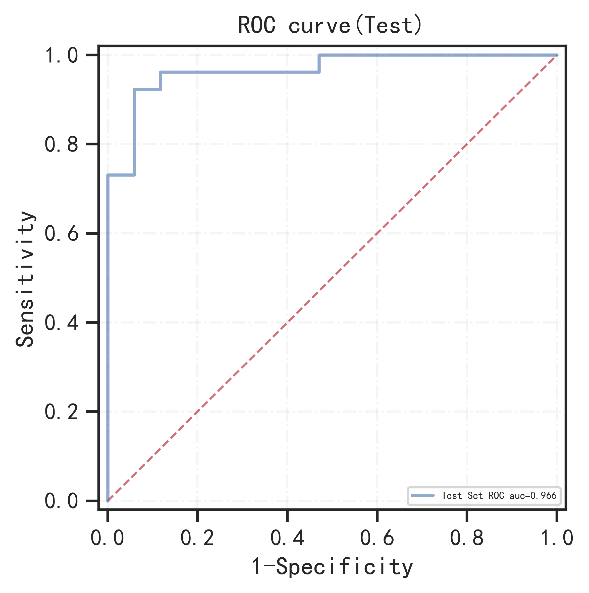

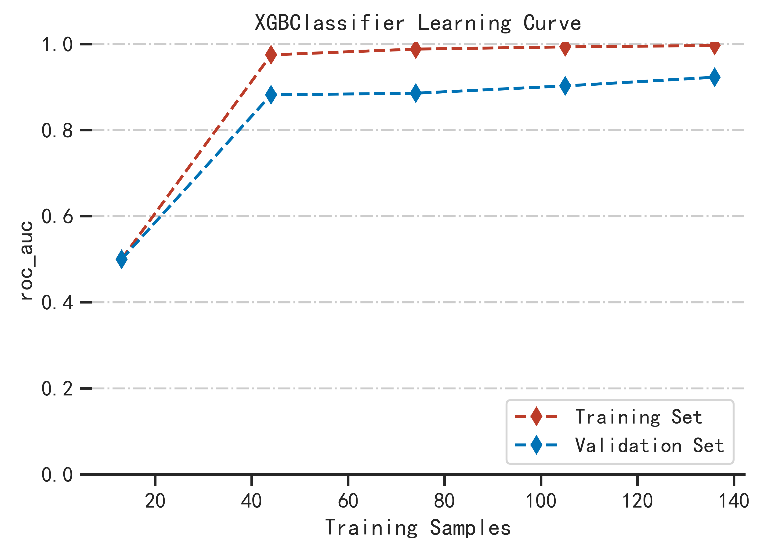


(c) (d)


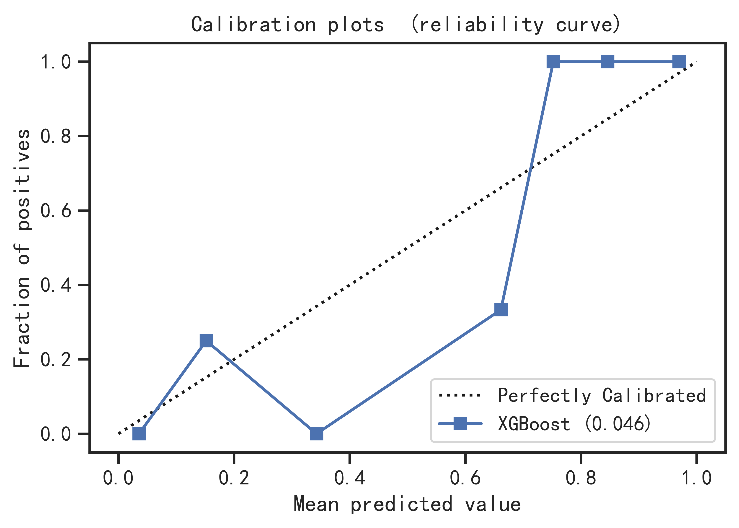

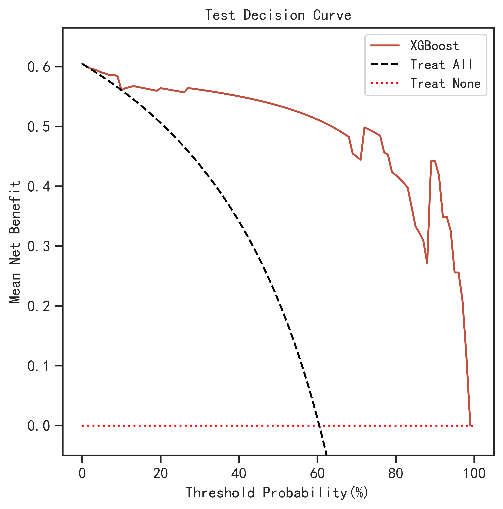


(e) (f)

**Supplementary Fig.S5:** Training, validation and testing of XGBoost model. The model parameters are: objective: binary: logistic; learning_rate: 0.3; max_depth: 8; min_child_weight: 2; reg_lambda (L2 regularization factor): 1. (a) Training set ROC and AUC; (b) Validation set ROC and AUC. training and cross-validation on 20% of patients with severe KOA. Solid lines of different colors represent 5 different results. (c) Test set ROC and AUC. results for 30% of patients with KOA. (d) Learning curve. The red dashed line represents the training set and the blue dashed line represents the validation set. (e) Calibration curves for the XGBoost model. (f) Decision analysis curves for predicting KOA progression model. The red dashed line represents the scenario that makes all patients mild KOA, and the black dashed line represents the inappropriate scenario that leads to progression in all patients. The solid red line represents the net benefit of the clinical application of the model.
